# Prognostic factors in pediatric head and neck rhabdomyosarcoma: a 30-year retrospective study in Brazil

**DOI:** 10.1590/1807-3107bor-2026.vol40.052

**Published:** 2026-08-21

**Authors:** Deisi Romitti Maglia, Bruna do Amaral Ferreira Souza, Taiane Berguemaier de Lima, Lauro José Gregianin, Fernanda Visioli

**Affiliations:** (a) Instituto do Câncer Infantil, Porto Alegre, RS, Brazil.; (b) Universidade Federal do Rio Grande do Sul – UFRGS, School of Dentistry, Department of Oral Pathology and Medicine, Porto Alegre, RS, Brazil.

**Keywords:** Rhabdomyosarcoma, Pediatrics, Prognosis, Therapeutics, Survival Analysis

## Abstract

This study aimed to evaluate the survival outcomes of pediatric patients with head and neck (HN) rhabdomyosarcoma (RMS) treated at a reference center in Brazil, comparing them with those of patients with non-head and neck (NHN) RMS. Additionally, it sought to identify the clinical-pathological variables, histological subtypes, and antineoplastic therapy regimens that significantly influence patient prognosis. The study included RMS patients treated between 1990 and 2022. Statistical analyses comprised t-tests, chi-square tests, Kaplan-Meier survival analysis, and Cox regression for overall survival (OS), disease-free survival (DFS), and event-free survival (EFS). A total of 64 patients (60.9% male) were included, with head and neck tumors being the most common presentation. Prognostic factors associated with OS included age ≥ 10 years, intermediate-risk classification, tumor relapse, and surgery. DFS was significantly influenced by age ≥10 years and high-risk classification, while EFS was associated with lesion size, histological subtype, and radiotherapy. In conclusion, anatomical site was not a significant prognostic factor. Age and risk classification significantly influenced OS and DFS, while histological subtype emerged as an independent factor for EFS. This study, conducted at a reference center in Latin America with a 30-year history of care, provides clinically relevant insights by identifying specific prognostic factors for RMS in Brazilian pediatric patients. These findings enable comparisons with international data and contribute to planning health interventions.

## Introduction

Rhabdomyosarcoma (RMS) is a rare neoplasm, primarily affecting children and adolescents. Despite this, it is considered the most common soft tissue sarcoma of childhood, accounting for approximately 3 to 4% of pediatric tumors diagnosed in the United States, mainly afflicting children in their first decade of life.^1,2^ RMS incidence is approximately 1.5 times higher in males, with a predilection for the head and neck (HN) anatomical site, followed by the genitourinary tract, extremities, and thorax.^3-5^ RMS originate from mesenchymal cells, which differentiate into primitive cells of striated muscles and can affect sites where this type of musculature is not present.^6^ Histologically, RMS is classified into four subtypes: embryonal (ERMS), alveolar (ARMS), spindle cell/sclerosing, and pleomorphic.^7^ Clinically, the tumor presents as a painless mass adhered to the musculature, growing rapidly and without a history of trauma. Signs and symptoms are typically local, nonspecific, and vary depending on the affected structures.^4,5,8^ When occurring in the HN region, RMS can be classified as either parameningeal (PM), non-parameningeal (non-PM), or orbital.^5^

The tumor's location is vital for prognosis assessment and guiding treatment. Planning involves staging, surgery, and risk assessment to tailor therapy.^3,9^ Treatment for RMS includes surgery, chemotherapy, and radiotherapy, either combined or alone.^9-13^

The compilation of information, including clinical staging, the use of multifaceted therapies, and continuous assessment through clinical trials, contributes significantly to improved survival rates among RMS patients.^1^ Factors like age, gender, tumor location, and subtype impact prognosis. These factors should be monitored and considered to better understand the epidemiological pattern of this tumor. This understanding informs new prognosis/risk classification strategies, facilitates the search for novel therapies, and devises ways to mitigate potential side effects of employed therapies, given their primary goal of enhancing the survival of patients.^2,9^ However, limited data exists for RMS patients in Latin America.

This study aimed to evaluate the survival of pediatric patients with HN RMS treated at a reference center in Brazil in comparison with non-head and neck (NHN) RMS. Additionally, we aimed to determine which clinical-pathological variables, histological subtypes, and employed antineoplastic therapy regimens significantly impact patient prognosis.

## Methods

This retrospective cohort study was conducted following the guidelines set forth by the STROBE Statement (Strengthening the Reporting of Observational Studies in Epidemiology). The present study received approval from the Research Ethics Committee on Human Beings of Hospital de Clínicas de Porto Alegre (HCPA, protocol CAAE 55719822.9.0000.5327) and from the Research Projects Committee of the Instituto do Câncer Infantil (No. 2022 – 0101). Informed consent was obtained from the participating individuals or their legal guardians.

**Figure 1:**
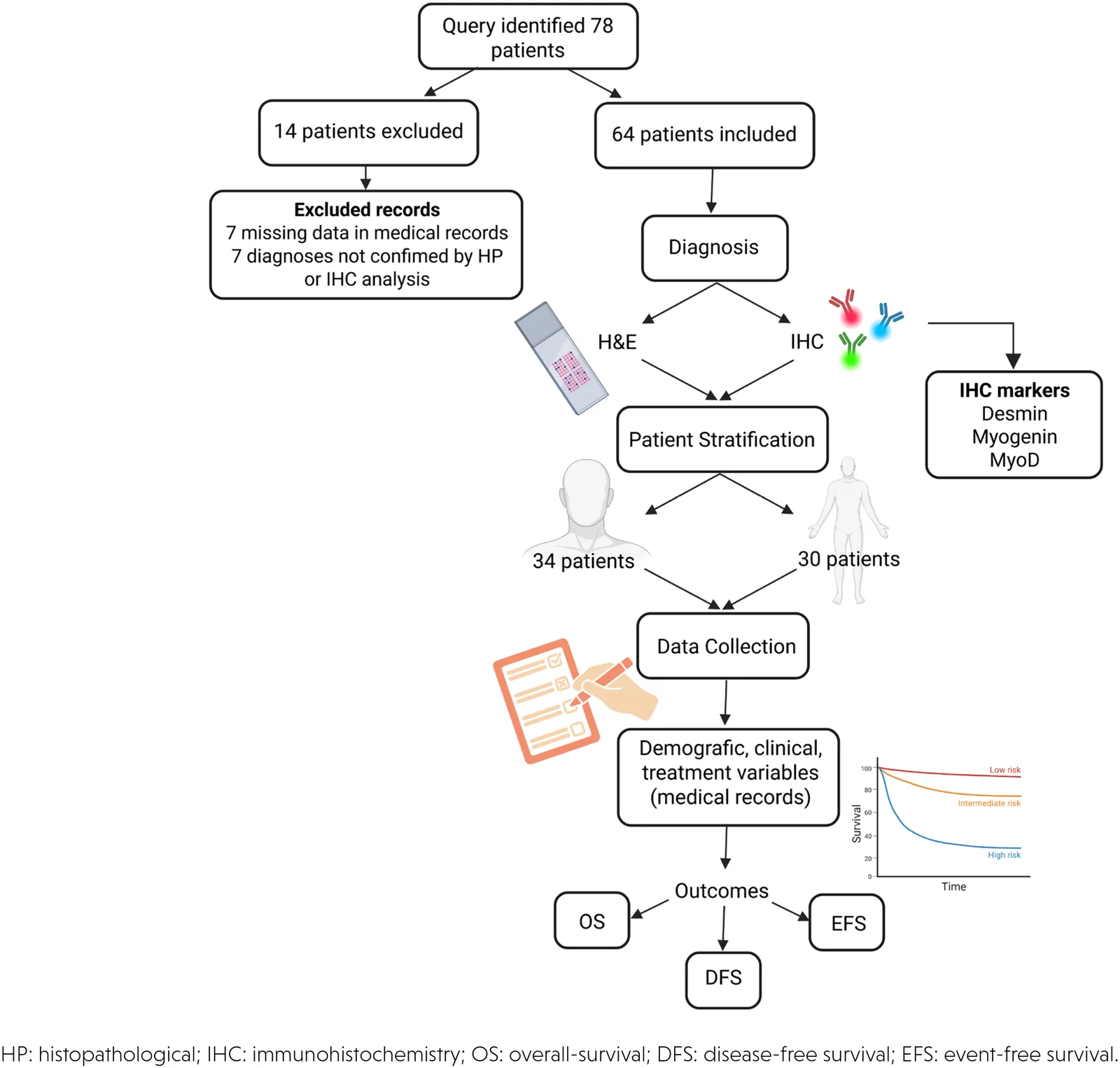
Flowchart of participant eligibility and study inclusion.

All individuals aged 0 to 19 years who were identified with lesions diagnosed as RMS (based on microscopic HE and immunohistochemistry analysis) between 1990 and 2022 were considered eligible for inclusion. Cases with insufficient information to confirm the diagnosis, as well as patients who underwent part of their treatment at another institution and for whom essential data could not be obtained, were excluded. The selection process and exclusion criteria are detailed in the flowchart (Figure 1). An immunohistochemical panel was performed in all selected cases, with the choice of markers varying depending on the case; however, muscle markers such as desmin, myogenin and MYOD1 were consistently included. Demographic, clinical, and treatment variables were obtained from medical records. Patient risk stratification was based on guidelines from the Children's Oncology Group (COG).^3^ Outcome variables related to overall survival (OS), disease-free survival (DFS), and event-free survival (EFS) were determined in months. OS was calculated considering the time interval between the date of diagnosis and the date of death or last medical follow-up. DFS was calculated from the time of primary tumor treatment completion to the date of recurrence or detection of regional or distant metastasis. Lastly, EFS was derived from the time interval between the date of diagnosis and the date of disease progression, recurrence, or death.

For statistical analysis, distribution normality was assessed through histograms and the Shapiro-Wilk test. Clinical-pathological variables were compared between patient groups using the chi-square test and Student's t-test. The influence of each variable on the outcome of OS, DFS, and EFS was initially assessed through univariable analysis and subsequently through multivariable Cox regression. Analyses were performed using SPSS 21.0 software. Participant survival analysis was demonstrated using the Kaplan-Meier method.

## Results

A total of 78 eligible patients were identified. Of these, 7 individuals were excluded due to the absence of information in the medical records, and another 7 were excluded because the histopathological or immunohistochemistry reports did not confirm the diagnosis of RMS. Finally, 64 patients diagnosed with RMS between 1990 and 2022 were included in the study (Figure 1). Among these, the majority were male (60.9%), with a mean age of 5.56 years (± 4.7) for patients with HN tumors, and 8.17 years (± 9.13) for patients with tumors in other body regions. The majority (54.6%) of primary tumors were larger than 5 cm (Table 1).

**Table 1:** Clinical and demographic characteristics of the sample.

Variables		Head and neck RMS (n = 34)	Non-head and neck RMS (n = 30)	p-value
		n (%)	n (%)	
Age at diagnosis in years (mean ± SD)		5.56 (± 4.70)	8.17 (± 9.13)	0.167
Sex				
	Male	21 (61.8)	18 (60.0)	0.885
	Female	13 (38.2)	12 (40.0)	
Race/Ethnicity				
	White	29 (85.3)	28 (93.3)	0.487
	Black	4 (11.8)	2 (6.7)	
	Indian	1 (2.9)	0 (0.0)	
Tumor size				
	< 5 cm	7 (30.4)	8 (29.6)	0.951
	> 5 cm	16 (69.6)	19 (70.4)	
TNM staging				
	Stage 1	12 (35.3)	5 (16.7)	0.255
	Stage 2	3 (8.8)	5 (16.7)	
	Stage 3	12 (35.3)	10 (33.3)	
	Stage 4	6 (17.6)	10 (33.3)	
	NI	1 (2.9)	0 (0.0)	
Risk stratification				
	Low risk	9 (26.5)	8 (26.7)	0.239
	Intermediate risk	18 (52.9)	11 (36.7)	
	High risk	6 (17.6)	9 (30.0)	
	NI	1 (2.9)	2 (6.7)	
Clinical classification				
	Group I	2 (5.9)	2 (6.7)	0.238
	Group II	2 (5.9)	5 (16.7)	
	Group III	23 (67.6)	12 (40.0)	
	Group IV	6 (17.6)	10 (33.3)	
	NI	1 (2.9)	1 (3.3)	
Histology				
	ERMS	22 (64.7)	19 (63.3)	0.216
	ARMS	8 (23.5)	11 (36.7)	
	Spindle cell/sclerosing	1 (2.9)	0 (0.0)	
	Unspecified	3 (8.8)	0 (0.0)	
Histological variant				
	Embrionary Botryoid	1 (11.1)	7 (38.9)	NA
	Alveolar Classic	5 (55.6)	10 (55.6)	
	Alveolar Solid	3 (33.3)	1 (5.6)	
Perineural invasion				
	Yes	1 (2.9)	1 (3.3)	0.839
	No	7 (20.6)	8 (26.7)	
	NI	26 (76.5)	21 (70.0)	
Vascular invasion				
	Yes	0 (0.0)	2 (6.7)	0.308
	No	8 (23.5)	7 (23.3)	
	NI	26(76.5)	21 (70.0)	
Surgery				
	Yes	12 (35.3)	16 (53.3)	0.255
	No	21 (61.8)	14 (46.7)	
	NI	1 (2.9)	0 (0.0)	
Tumor-free surgical margins				
	Yes	4 (33.3)	8 (50.0)	0.407
	No	5 (41.7)	3 (18.8)	
	NI	3 (25.0)	5 (31.3)	
Chemotherapy				
	Yes	34 (100.0)	30 (100.0)	NA
	No	0 (0.0)	0 (0.0)	
Radiotherapy				
	Yes	32 (94.1)	16 (53.3)	0.000
	No	2 (5.9)	14 (46.7)	
Total dose of radiation				
Mean (± SD)		46.88 (±7.96)	44.64 (±3.47)	0.104
Disease recurrence				
	Yes	7 (20.6)	8 (26.7)	0.567
	No	27 (79.4)	22 (73.3)	
Node metastasis at diagnosis				
	Yes	6 (17.6)	13 (43.3)	0.025
	No	28 (82.4)	17 (56.7)	
Distant metastases at diagnosis				
	Yes	7 (20.6)	9 (30.0)	0.386
	No	27 (79.4)	21 (70.0)	
Late node metastasis				
	Yes	4 (11.8)	3 (10.0)	0.821
	No	30 (88.2)	27 (90.0)	
Late distant metastasis				
	Yes	4 (11.8)	7 (23.3)	0.221
	No	30 (88.2)	23 (76.7)	
Death				
	Yes	13 (38.2)	13 (43.3)	0.679
	No	21 (61.8)	17 (56.7)	
Overall survival (mean ± SD)		158.79 ± 123.20	143.43 ± 123.29	0.869
Disease-free survival (mean ± SD)		140.88 ± 126.06	109,13 ± 124,07	0.952
Event-free survival(mean ± SD)		154.26 ± 127.42	121.30 ± 125.38	0.935

CP: RMS in the head and neck region; NCP: RMS in regions other than the head and neck; NA: Not applicable; NI: Not informed.

**Figure 2:**
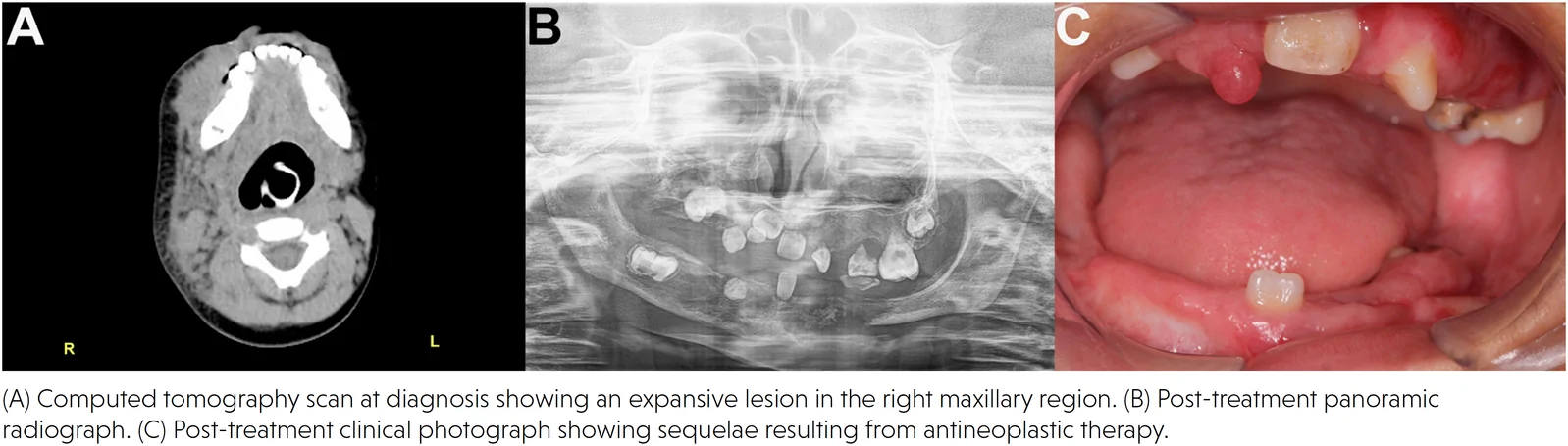
Representative images of a rhabdomyosarcoma case in the right cheek.

Among patients with tumors in the HN region, the majority were located in the PM region (n = 16, 47%), with the most affected site being the nasal cavity (n = 3, 18.8%). Among the 7 patients with non-PM lesions, the most affected site was the cheek (42.9%), as illustrated in a representative case of HN RMS (Figure 2). Additionally, 11 patients were diagnosed with RMS in the orbital region. Among the remaining patients (n = 30) with RMS in other body locations, the most affected site was the bladder (20%), followed by lesions in the lower limbs (16.7%) (see Table 2 for a detailed anatomical distribution of tumor sites).

**Table 2:** Anatomical distribution of primary tumor sites in patients with RMS.

Variables		Frequency (n)	Percentage (%)
Primary tumor location – Parameningeal			
	Middle ear	1	1.6
	Nasal cavity	3	4.7
	Paranasal sinuses	2	3.1
	Nasopharynx	2	3.1
	Infratemporal fossa	1	1.6
	Pterygopalatine area	1	1.6
	Parapharyngeal area	1	1.6
	Other	5	7.8
	Total	16	25
Primary tumor location – Non-parameningeal (HN)			
	Cheek	3	4.7
	Neck	1	1.6
	Other	3	4.7
	Total	7	10.9
Primary tumor location – Orbital			
	Right orbit	5	7.8
	Left orbit	5	7.8
	Left eyelid	1	1.6
	Total	11	17.2
Primary tumor location – Regions outside head and neck			
	Right upper limb	2	3.1
	Left upper limb	2	3.1
	Right lower limb	5	7.8
	Left lower limb	2	3.1
	Bladder	6	9.4
	Anus	1	1.6
	Testicle	4	6.3
	Prostate	2	3.1
	Uterus	1	1.6
	Retroperitoneum	1	1.6
	Biliary tract	1	1.6
	Other	3	4.7
	Total	30	46.9
**TOTAL**		**64**	**100**

ERMS was the most prevalent histological subtype for both HN and non-HN RMS, accounting for 64.7 and 63.3%, respectively (Table 1). Regarding the TNM clinical staging system, 18 patients with HN RMS were at stage 3 and 4, representing 52.9% of the studied sample; for patients with non-HN RMS, this percentage increased to 66.6% of individuals. Regarding risk stratification, out of the 64 patients included in the study, 52.9% were classified as intermediate risk, for both HN and non-HN RMS patients, and 36.7% were classified as high risk. Among patients classified as high risk, 53.3% were diagnosed with ARMS (Table 1).

All patients included in the study underwent at least one chemotherapy (CT) regimen (Table 1). CT regimens varied according to the risk classification, as shown in Table 3. Radiotherapy (RT) was more frequently performed in HN RMS (p < 0.001). For the majority of patients with HN RMS, surgery was not indicated for treatment (63.3%); conversely, 53.3% of patients with non-HN RMS underwent complete tumor resection. The time interval between diagnosis and initiation of the first treatment was of 9.18 days (± 23.40). The mean interval was 8.45 days (± 24.00) for patients with non-HN RMS and 9.82 days (± 23.21) for those with HN RMS. Out of a total of 64 patients included in the study, 26 died (40.6%). There was no significant difference in the mean OS, DFS, and EFS comparing HN and non-HN patients (Table 1).

**Table 3:** Chemotherapy regimens administered according to risk stratification

Variables		Risk stratification			
		Low risk	Intermediate risk	High risk	NI
Chemotherapy regimen 1					
	VA	6	0	0	0
	VAC	9	17	4	0
	VAC/IE	0	1	3	0
	VAC/VDC	1	1	0	0
	VAC/VDC/IE/VI	0	4	1	1
	VAC/VI	0	1	0	0
	VC	0	0	1	0
	Others	1	4	6	2

VA: Vincristine and Actinomycin D; VAC: Vincristine, Actinomycin D, and Cyclophosphamide; VAC/IE: Vincristine, Actinomycin D, Cyclophosphamide, Ifosfamide, Etoposide; VAC/VDC/IE/VI: Vincristine, Actinomycin D, Cyclophosphamide, Doxorubicin, Ifosfamide, Etoposide, Irinotecan; VAC/VI: Vincristine, Actinomycin D, Cyclophosphamide, Irinotecan; VC: Vincristine, Cyclophosphamide.

**Figure 3:**
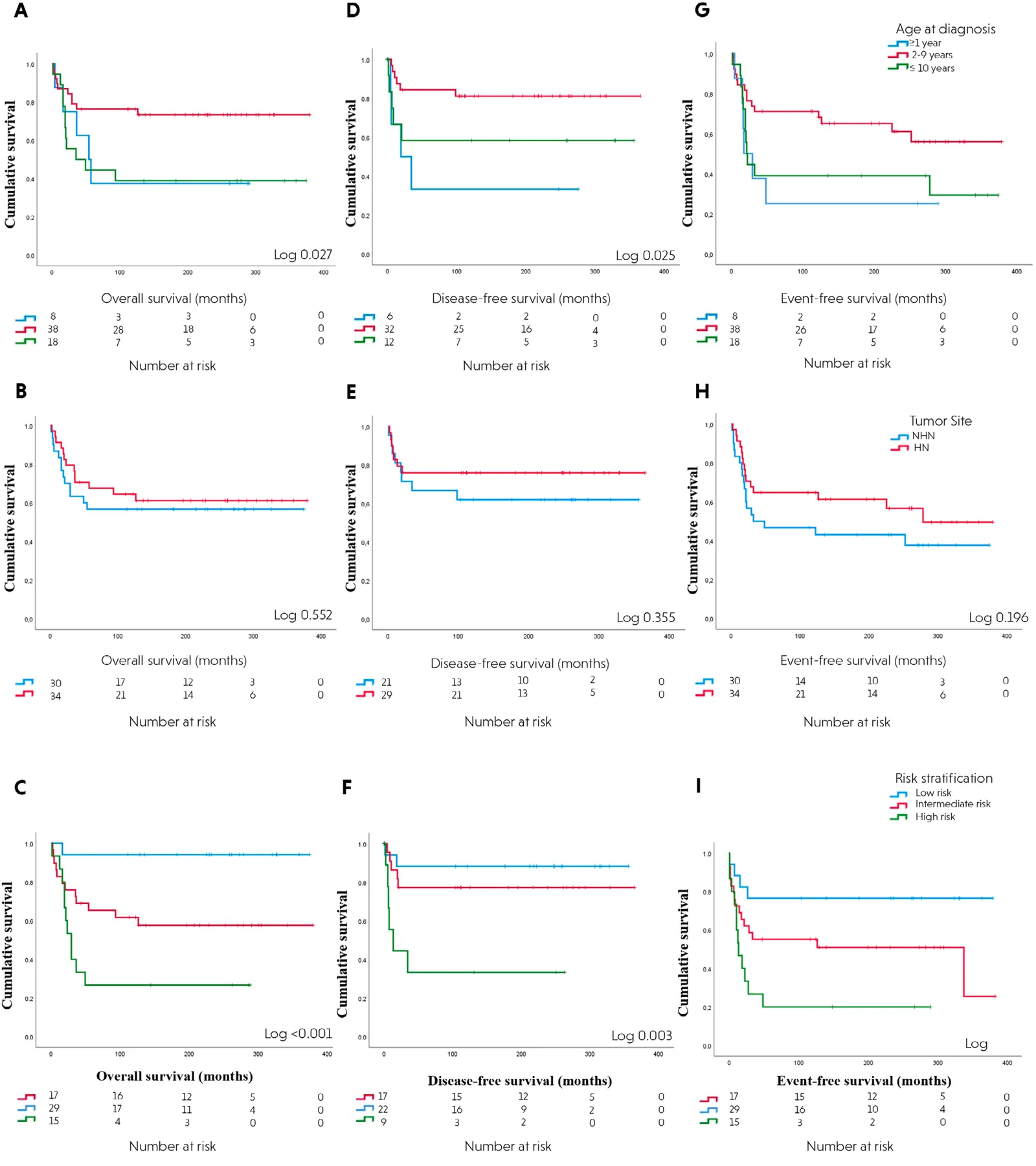
Overall survival (A, B, C), disease-free survival (D, E, F), and event-free survival (G, H, I) curves according to clinical variables.

**Figure 4:**
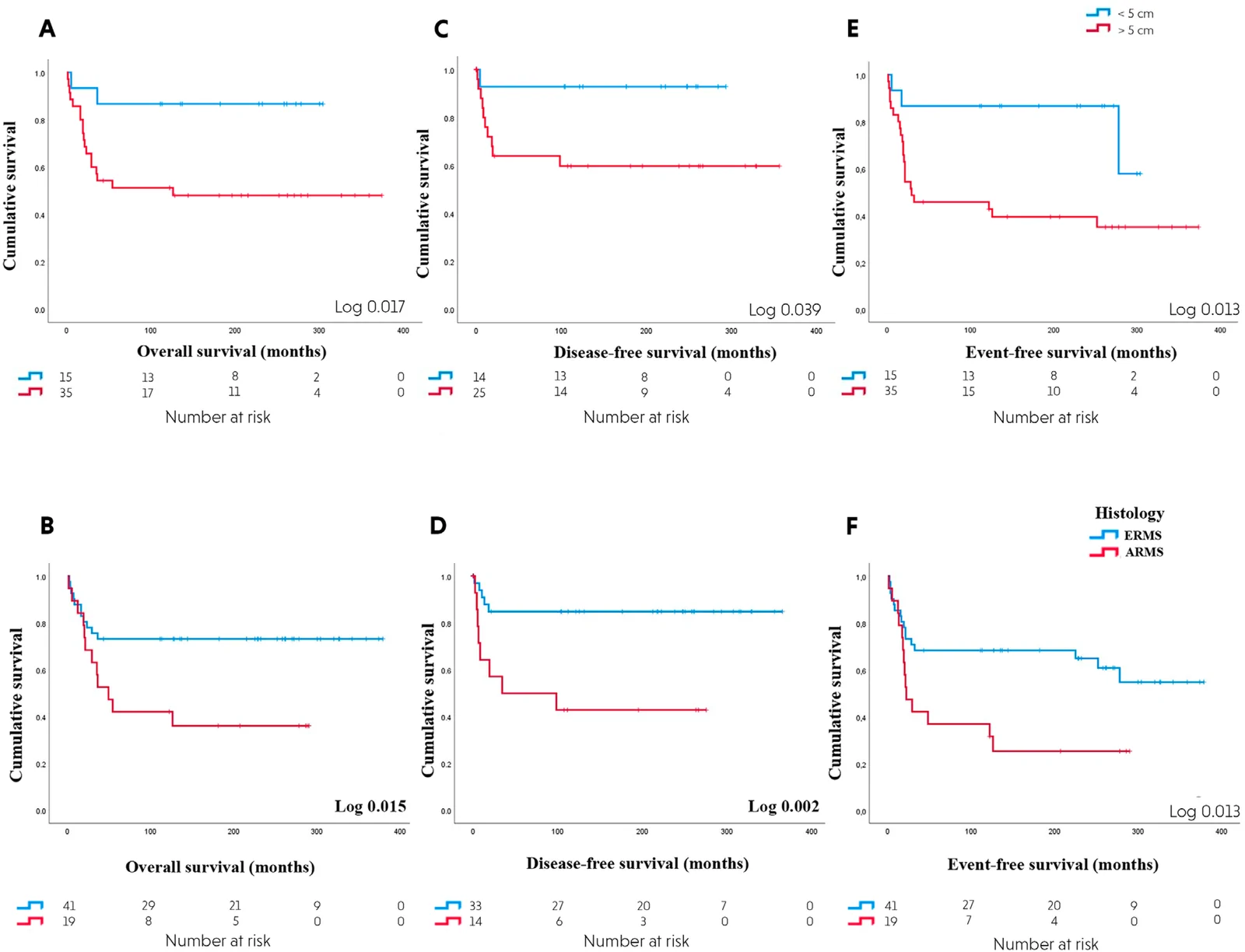
Overall survival (A, B), disease-free survival (C, D), and event-free survival (E, F) curves according to clinical-pathological variables.

The Kaplan-Meier survival analysis revealed statistically significant differences in OS, DFS, and EFS among patients based on lesion size > 5 cm, clinical staging, clinical group, risk classification, distant metastasis at diagnosis, lesion location, and histological subtype. The age subgroup showed statistical significance only for OS and DFS. Variables related to the development of regional and distant metastases after treatment initiation were statistically significant only for OS. Regarding treatments, undergoing surgery was statistically a protective factor in OS assessment; RT significantly improved EFS, while (QT) significantly influenced patients’ OS. The specific Kaplan-Meier curves are depicted in Figures 3 and 4.

The Cox univariate analysis related to OS indicates that age ≥10 years at diagnosis (HR: 2.890; 95%CI: 1.222–6.835), disease recurrence (HR: 2.482; 95%CI: 1.131–5.446), tumors measuring more than 5 cm at diagnosis (HR: 4.967; 95%CI: 1.150–21.456), and stage 4 tumors (HR: 6.040; 95%CI: 1.673–21.798) increased the likelihood of the patient progressing to death. Intermediate-risk (HR: 8.796; 95%CI: 1.143–67.693) and high-risk (HR: 19.919; 95%CI: 2.553–155.380) classifications increased the odds of death by 8.7 and 19.9 times, respectively. The histological subtype ARMS presented a 2.6 times higher chance of death than ERMS (HR: 2.630; 95%CI: 1.158–5.974). In the therapeutic context, surgery was a protective factor, reducing the risk of death by 63% (HR: 0.377; 95%CI: 0.157–0.904), while non-traditional chemotherapy protocols increased the odds of death (HR: 2.697; 95%CI: 1.203–6.047) (Table 4). The multivariate analysis with adjusted models confirmed age ≥ 10 years (HR: 18.409; 95%CI: 4.049-83.698) and recurrence (HR: 5.256; 95%CI: 1.194–23.143) as independent risk factors for OS, increasing the risk of death by 18 times and 5.2 times, respectively. Intermediate-risk classification increased the odds of death by 9.6 times (HR: 9.679; 95%CI: 1.060–88.378), while surgery was a significant independent protective factor (HR: 0.121; 95%CI: 0.024–0.611). The histological subtype did not impact OS in the multivariate analysis (Table 5).

**Table 4:** Univariate Cox analyses for overall survival (OS), disease-free survival (DFS), and event-free survival (EFS).

Variables		Overall survival						Disease-free survival						Event-free survival					
		n (%)	Mean OS	SD	HR	95%CI	p-value	n (%)	MEAN DFS	SD	HR	95%CI	p-value	n (%)	Mean EFS	SD	HR	95%CI	p-value
Decade																			
	1990–2000	14	280.71	115.57	ref		0.150	13	266.62	114.33	ref		0.422	14	263.14	132.02	ref		.041
	2000–2010	33	133.27	112.05	4.31	0.99–18.77	0.051	24	150.63	108.59	2.790	0.60–12.92	0.190	33	121.09	115.553	5.04	(1.43 - 17.67)	.011
	> 2010	17	81.71	51.41	3.80	0.80–17.97	0.091	13	75.62	51.65	2.203	0.40–12.04	0.362	17	70.82	55.79	4.23	(1.07 - 16.72)	.039
Age at diagnosis (years)																			
	≤ 1	8	125.75	128.94	2.68	0.91-7.85	0.072	6	97.33	127.55	4.769	1.34–16.97	0.016	8	85.00	118.53	2.55	(.98 - 6.60)	.054
	2–9	38	170.21	113.17	Ref		0.033	32	175.47	107.27	ref		0.042	38	159.79	118.96	ref		.073
	≥ 10	18	124.61	137.91	2.89	1.22-6.83	0.016	12	155.42	145.53	2.779	0.84–9.11	0.092	18	118.44	141.35	2.05	(.95 - 4.40)	.065
Disease recurrence																			
	No	49	172.61	125.77	Ref														
	Yes	15	83.93	082.66	2.48	1.13–5.44	0.023	NA	NA	NA	NA	NA	NA	NA	NA	NA	NA	NA	NA
Tumor site																			
	HN favorable	18	178.83	110.79	Ref		0.536	18	157.33	117.47	ref		0.513	18	171.28	119.78	ref		.202
	HN unfavorable	16	136.25	135.85	1.74	0.58–5.20	0.318	11	178.00	130.60	.617	0.12–3.18	0.565	16	135.13	136.81	1.19	(.43 - 3.30)	.729
	NHN favorable	7	139.86	96.69	0.93	0.18–4.64	0.937	6	147.17	92.91	.594	0.06–5.09	0.635	7	140.14	96.34	.67	(.14 - 3.18)	.620
	NHN unfavorable	23	145.17	132.22	1.89	0.70–5.13	0.208	15	159.40	133.49	1.676	0.53–5.28	0.378	23	115.57	126.55	2.08	(.89 - 4.88)	.090
Tumor size > 5 cm																			
	No	15	190.12	95.78	Ref			14	190.14	85.96	ref			15	188.87	98.06	ref		
	Yes	35	127.00	125.01	4.96	1.15–21.45	0.032	25	141.36	127.94	6.548	0.83–51.23	0.073	35	114.31	127.69	4.10	(1.22 - 13.75)	.022
TNM Staging System																			
	Stage 1	17	227.24	106.90	Ref		0.015	17	208.82	112.39	ref		0.019	17	222.47	114.73	ref		.007
	Stage 2	8	173.25	84.04	.68	0.07–6.61	0.745	8	158.00	89.58	.648	.06–6.23	0.707	8	170.13	89.40	.45	(.05 - 3.86)	.466
	Stage 3	22	137.27	130.74	3.46	0.95–12.60	0.059	14	174.00	130.07	1.214	.24–6.01	0.813	22	122.18	134.92	2.90	(1.03 - 8.17)	.043
	Stage 4	16	86.94	107.27	6.04	1.67–21.79	0.006	10	80.90	102.42	5.445	1.39–21.19	0.015	16	64.88	91.08	4.84	(1.70 - 13.78)	.003
Risk stratification																			
	Low risk	17	237.24	97.06	Ref		0.007	17	218.00	104.37	ref		0.011	17	229.82	109.88	ref		.009
	Intermediate risk	29	145.07	120.28	8.79	1.14–67.69	0.037	22	161.09	117.51	1.992	0.38–10.27	0.410	29	134.21	124.75	2.67	(.87 - 8.16)	.083
	High risk	15	82.27	106.93	19.91	2.55–155.38	0.004	9	79.00	108.45	8.334	1.66–41.60	0.010	15	62.87	92.05	5.66	(1.79 - 17.84)	.003
Node metastasis at diagnosis																			
	No	45	168.33	122.85	ref			36	181.81	117.47	ref			45	157.82	129.86	ref		
	Yes	19	112.74	115.44	1.80	0.81–3.97	0.147	14	108.50	112.77	2.821	1.02–7.79	0.045	19	93.79	108.58	1.90	(.94 - 3.84)	.072
Distant metastases at diagnosis																			
	No	48	173.75	120.13	Ref			40	181.37	116.27	ref			48	163.75	127.47	ref		
	Yes	16	86.06	107.90	3.18	1.45–6.97	0.004	10	80.90	102.42	4.715	1.69–13.08	0.003	16	64.00	91.60	2.98	(1.46 - 6.06)	.003
Histology																			
	ERMS	41	177.95	125.41	Ref			33	195.91	113.83	ref			41	163.75	127.47	ref		
	ARMS	19	108.37	110.40	2.63	1.15 -5.97	0.021	14	99.50	107.42	4.798	1.56–14.71	0.006	19	87.16	103.58	2.42	(1.17 - 4.99)	.017
Radiotherapy																			
	No	16	113.94	116.07	Ref			9	145.89	105.07	ref			16	91.06	109.56	ref		
	Yes	47	164.46	123.12	0.54	0.23–1.25	0.156	41	164.66	123.64	.882	0.24–3.12	0.846	47	154.73	128.85	.43	(.20 - .89)	.024
Surgery																			
	No	35	118.83	117.84	Ref			23	145.78	115.153	ref			35	108.51	116.57	ref		
	Yes	28	198.21	114.50	0.37	0.15–.90	0.029	27	174.48	124.11	1.367	0.48–3.84	0.553	28	181.36	128.67	.68	(.33 - 1.39)	.297
Tumor-free surgical margins																			
	Yes	12	189.08	117.56	Ref			12	152.25	129.66	ref			12	165.33	130.41	ref		
	No	8	241.75	113.86	0.55	0.16–5.74	0.962	8	194.63	124.27	.571	0.11–2.94	0.504	8	209.00	123.82	.96	(.25 - 3.65)	.963
Chemotherapy																			
	VAC, VA	36	175.97	114.24	Ref		0.026	32	171.69	115.89	ref		0.454	36	163.44	123.70	ref		.131
	VAC/VDC/IE/VI	6	123.83	45.22	0.49	0.06–3.79	0.495	6	96.67	38.60	.525	0.06–4.14	0.541	6	114.00	35.72	.68	(.15 - 2.99)	.616
	Others	21	125.29	144.40	2.69	1.20–6.04	0.016	12	165.83	150.71	1.712	0.57–5.11	0.336	21	109.90	142.63	1.93	(.94 - 3.98)	.073

HN favorable: Head and Neck favorable; HN unfavorable: Head and Neck unfavorable; NHN favorable: Non-Head and Neck favorable; NHN unfavorable: Non-Head and Neck unfavorable; ERMS: Embryonal Rhabdomyosarcoma; ARMS: Alveolar Rhabdomyosarcoma; VA: vincristine and actinomycin D; VAC: vincristine, actinomycin D, and cyclophosphamide; VAC/VDC/IE/VI: vincristine, actinomycin D, cyclophosphamide, doxorubicin, ifosfamide, etoposide, irinotecan.

**Table 5:** Multivariate Cox analyses for overall survival (OS), disease-free survival (DFS), and event-free survival (EFS).

Variables			HR	95%CI	p-value
Overall survival					
	Age at diagnosis (years)				
		≤ 1	2.51	0.49–12.75	0.266
		2–9	ref		0.001
		≥ 10	18.40	4.04–83.69	0.000
	Disease recurrence				
		No	ref		
		Yes	5.25	1.19–23.14	0.028
	Risk stratification				
		Low risk	ref		0.106
		Intermediate risk	9.67	1.06–88.37	0.044
		High risk	5.98	0.51–69.25	0.152
	Histology				
		ERMS	ref		
		ARMS	.54	0.16–1.74	0.304
	Surgery				
		No	ref		
		Yes	0.12	0.02–0.61	0.011
Disease-free survival					
	Age at diagnosis (year)				
		≤ 1	3.25	0.76–13.83	0.110
		2–9	ref		0.020
		≥ 10	6.49	1.68–25.03	0.007
	Risk stratification				
		Low risk	ref		0.005
		Intermediate risk	3.48	0.63–19.23	0.152
		High risk	15.83	2.64–94.79	0.002
Event-free survival					
	Decade				
		1990–2000	ref		0.212
		2000–2010	7.12	0.77–65.58	0.083
		> 2010	7.62	0.73–79.04	0.089
	Tumor size > 5 cm				
		No	ref		
		Yes	7.82	1.85–33.01	0.005
	Node metastasis at diagnosis				
		No	ref		
		Yes	0.38	0.12–1.17	0.095
	Distant metastases at diagnosis				
		No			
		Yes	2.92	1.08–7.86	0.034
	Histology				
		ERMS	ref		
		ARMS	4.38	1.29–14.87	0.018
	Radiotherapy				
		No	ref		
		Yes	0.08	0.02–.36	< 0.001

ERMS: Embryonal rhabdomyosarcoma. ARMS: Alveolar rabdomyosarcoma.

Regarding DFS, the Cox univariate analysis reveals that age ≤1 year (HR: 4.769; 95%CI: 1.340–16.977), stage 4 disease (HR: 5.445; 95% CI: 1.399–21.192), high-risk classification (HR: 8.334; 95%CI: 1.699–41.605), and the presence of regional (HR: 2.821; 95%CI: 1.021–7.792) and distant metastasis (HR: 4.715; 95%CI: 1.699–13.083) at diagnosis significantly impacted DFS. ARMS increases the chances of recurrence by 4.7 times (HR: 4.798; 95%CI: 1.565–14.711) (Table 4). Conversely, the multivariable Cox analysis for DFS showed that the age group ≥10 years at diagnosis had a higher chance of disease recurrence (HR: 6.495; 95%CI: 1.685–25.038), while age ≤1 year was no longer statistically relevant. Finally, risk classification reinforced that high-risk patients have a lower DFS, with 15.8 times more chances of tumor recurrence (HR: 15.838; 95%CI: 2.646–94.798) (Table 5).

The Cox univariate analysis for EFS revealed that age did not influence the patients’ EFS time. Lesion size (HR: 4.107; 95% CI: 1.226–13.754) and stages 3 (HR: 2.906; 95%CI: 1.034–8.170) and 4 tumors (HR: 4.844; 95%CI: 1.702–13.788) demonstrated a significant influence on EFS. The probability of disease progression was higher for patients classified as high risk (HR: 5.662; 95%CI: 1.797-17.845) and with distant metastasis at diagnosis (HR: 2.985; 95% CI: 1.468–6.068), resulting in a lower EFS. The histological subtype ARMS increased the chance of disease progression by 2.4 times (HR: 2.420; 95%CI: 1.173–4.995). Regarding treatment types, patients undergoing radiotherapy (RT) had a lower probability of disease progression (HR: 0.431; 95%CI: 0.207–0.896) (Table 4). The multivariate analysis of EFS indicated that lesions larger than 5 cm (HR: 7.829; 95%CI: 1.856–33.014), the presence of distant metastasis at diagnosis (HR: 2.923; 95%CI: 1.087–7.860), and the histological subtype ARMS (HR: 4.381; 95%CI: 1.290–14.877) were independent risk factors associated with an increased probability of disease progression. On the other hand, the administration of RT was a protective factor for EFS (HR: 0.087; 95%CI: 0.021–0.365) (Table 5).

## Discussion

RMS is a malignant tumor originating from immature skeletal muscle cells and typically affects children and adolescents.^1^ Several international studies have assessed the OS, DFS and EFS of the affected population using population-based cancer registries.^2-4^ Understanding the profile of patients affected by this neoplasm in different geographical centers is crucial for establishing prognostic factors and planning health interventions. Our study conducted a survey of RMS patients treated at a reference center in Brazil from 1990 to 2022, representing the largest Brazilian cohort published to date. The aim was to evaluate and compare the OS, DFS, and EFS of this population with studies conducted in other research centers. Additionally, we aimed to determine which clinical-pathological variables, histological subtypes, and antineoplastic therapy regimens impact the prognosis of RMS patients.

The socio-demographic analysis revealed a predominance of male patients, white ethnicity, aged under 10 years, with a higher incidence in the HN region, and ERMS as the most frequent histological type. These findings align with the results from various studies conducted in North American,^3,4^ European,^5^ and Asian populations.^6-8^ Similarly, a recent Latin American multicenter study by Gallagher et al. (2024) reported ERMS as the predominant histological subtype and that head and neck RMS accounts for approximately 35% of pediatric RMS cases, with particular involvement of the oral cavity and sinonasal regions.^14^

In the present study, the location of the tumor was not an independent risk factor for patient survival. Although tumors are more commonly observed in the HN region, the orbital and non-PM anatomical regions are not considered independent prognostic factors for OS, consistent with the findings of Wu and Zeng.^15^ As highlighted by Merks et al.^16^ and more recently by Schoot et al.,^17^ while the PM region may constitute an adverse prognostic factor, patient survival can be substantially influenced by effective lesion management through localized therapies, primarily RT. In our study, 94.1% of patients with HN RMS underwent RT, which probably positively impacted the prognosis of these patients. The anatomical location of RMS directly impacts therapeutic options. Tumors arising in surgically challenging sites, such as the PM region and genitourinary tract, pose a significant clinical challenge due to the proximity of critical structures and anatomical complexity, which limit the feasibility of radical interventions and increase the risk of functional and aesthetic sequelae.^11-13,18^ In such cases, RT becomes a cornerstone for local control, often integrated into intensified systemic regimens. In contrast, orbital tumors and those located in more accessible HN sites are usually diagnosed earlier, allowing for less mutilating therapeutic approaches and better functional preservation.^18,19^

Among the assessed variables, age and risk classification emerged as important risk factors for patient prognosis, impacting OS and DFS in both univariate and multivariate analysis. This finding was similar to a North American population with ERMS, as demonstrated by Wang et al.^20^ However, a recent analysis of the SEER public database comprising 446 cases revealed that primary site in parameningeal region, alveolar RMS histology, M1 stage, stage 4, surgery, and chemotherapy were significant prognostic factors.^15^ These contradictory findings reinforce the importance of performing prognosis analysis for each geographic population.

According to the findings in the present study, patients aged ≤ 1 year and ≥ 10 years at the time of diagnosis had a lower mean OS and DFS than patients aged between 2 and 9 years. Recent research has also reinforced this trend of a poorer treatment response at the extremes of age.^1-3^ For Rees et al.,^3^ patients under 1 year of age at the time of diagnosis pose a challenge related to antineoplastic therapies as they are more susceptible to acute and late effects, resulting in significant variations in the administered treatment. Meanwhile, Ferrari et al.^21^ suggest that the lower survival of adolescent patients may be attributed, among other factors, to differences in tumor biology, delays in the diagnostic process, and lower participation in clinical trials compared to younger children.

Risk classification, which is related to clinical staging (TNM), clinical group (based on disease extent), and histological subtype^6^, proved to be a significant determinant in OS and DFS but it was not an independent factor for EFS. In this study, the OS for patients with a high-risk classification averaged 82 months, DFS averaged 79 months, while EFS was 62 months. In contrast, the OS, DFS, and EFS for low-risk patients exceeded 200 months. These findings are consistent with prior literature, demonstrating that patients with metastatic disease (regional and distant) have a worse prognosis compared to those with localized disease.^1,3^

The most common histological subtype in the analyzed cohort was ERMS. Moreover, similar to Markiz et al.,^6^ this cohort showed that the ARMS histological subtype had a higher number of disease recurrences and a greater number of patients with high-risk disease. Although in the univariate analysis the ARMS histological subtype significantly impacted OS, DFS, and EFS, in the multivariate analysis, the histological subtype was confirmed as an independent risk factor only for EFS. The study by Oberlin et al.^22^ showed that histological subtype was strongly dependent on other variables such as age and staging. Conversely, Rees et al.^3^ showed that children diagnosed with ARMS have twice the chances of dying compared to patients diagnosed with ERMS. ARMS, which has been associated with a worse prognosis, is characterized by a chromosomal translocation involving the fusion of FOXO1 and PAX3 or PAX7 transcription factor genes.^9^ Totadri et al.^10^ argued that the fusion status can be decisive in risk stratification and treatment-related decisions, and therefore, fusion testing should be performed in patients diagnosed with ARMS or mixed or unspecified RMS histology. As a limitation, our study did not perform an analysis of PAX-FOXO1 translocations due to the unavailability of this test at the evaluated reference center, primarily because of its high cost. However, our results underscore the significance of other clinical-pathological variables for risk assessment. These variables should be thoroughly evaluated in healthcare facilities where molecular analyses are not yet feasible.

Treatment for RMS is multimodal and tailored to risk, always aiming to reduce morbidities associated with local disease control.^11,12^ Our multivariate analysis showed that CT and RT did not impact OS and DFS. Regarding EFS, RT significantly contributed to preventing disease progression, as did surgery, which acted as an independent protective factor for OS. Studies by Dombrowski et al.^13^ and Wu et al.^2^ revealed that surgery is significantly relevant for the treatment of RMS in the HN region and in the pelvic and genitourinary region, respectively. However, complete tumor resection depends on factors such as its location, size, and impact on form and function^11-13^. In some cases, the use of induction CT may reduce the tumor, facilitating surgical resection and reducing the doses of RT administered.^12^.

CT protocols used can vary according to different international cooperation groups (such as the Children's Oncology Group and the European Pediatric Soft Tissue Sarcoma Study Group) and based on the patients’ risk classification. Generally, an alkylating agent (cyclophosphamide or ifosfamide) is combined with vincristine and dactinomycin.^9,11^ In the present analysis, the VAC chemotherapy regimen (vincristine, actinomycin-D, cyclophosphamide) was the most commonly used, regardless of the risk classification. According to studies, the VAC chemotherapy regimen is commonly favored in low and middle-income countries due to its convenience of administration in an outpatient setting, especially applicable to patients with intermediate and high-risk RMS.^10^ Despite refinements in risk-adapted therapy, long-term outcomes remain poor for patients with metastatic or refractory disease, with survival rates below 30%.^23-25^ While VAC and isofasfamide, vincristine, and actinomycin D (IVA) regimens have been widely adopted for decades, improvements have been largely limited to adjustments in dose, schedule, and route of administration.^25^ Intensified regimens, such as carboplatin, epirubicin, vincristine, dactinomycin, ifosfamide, and etoposide (CEVAIE), have not shown superior efficacy in high-risk RMS.^24^ These findings highlight the urgent need for more effective therapeutic strategies, including novel targeted therapies and immunotherapeutic approaches, particularly for patients with metastatic or relapsed disease.^25^

RT can be employed for the treatment of the primary lesion, control of residual disease, or for metastatic lesions.^12,13,27^ Typically, doses range from 36 Gy to 55.8 Gy at the primary lesion site.^28^ In the current analysis, 48 patients underwent RT, and patients with RMS in the HN region were significantly more subjected to this therapy compared to patients with RMS in other sites. In the overall analysis, there was no increase in OS and DFS related to the administration of RT. Conversely, in the multivariate analysis of EFS, RT was confirmed as a protective factor against disease progression. As highlighted by Mohan et al.,^29^ 5-year OS and EFS were higher in patients undergoing RT and/or surgery. However, contradicting the results of the present study, Cameron et al.^27^ have not detected RT as an independent risk factor for EFS, only for OS.

The limitations of this investigation stem from its retrospective design, relying on the analysis of medical records. Furthermore, the study's observation period included the implementation of varying treatment protocols over the years, which added complexity to the analysis. Despite the relatively small sample size, our multivariate analysis identified independent prognostic factors with a significant impact on patient outcomes, reinforcing current knowledge of RMS prognosis and underscoring the importance of risk-adapted, multimodal strategies.

## Conclusions

According to the analyses conducted in this study, it was concluded that the majority of Brazilian patients with RMS were male, aged between 2 and 9 years, HN region was the most affected location, and ERMS was the most common histological subtype. As prognostic factors in a Brazilian population, age and risk classification had a significant impact on OS and DFS but did not influence EFS. Disease recurrence was a risk factor for OS. Histological subtype was confirmed as an independent risk factor for EFS. Regarding treatments, undergoing surgery was associated with increased OS, acting as a protective factor, and RT was considered an independent protective factor for EFS.

## Data Availability

The datasets generated during and/or analyzed during the current study are available from the corresponding author on reasonable request.
